# Case of Extra Uterine Pregnancy, Continuing Three Years and Six Months—Fœtus Removed by Gastrotomy

**Published:** 1860-03

**Authors:** C. Goodbrake

**Affiliations:** Clinton, Ill.


### THE CHICAGO MEDICAL JOURNAL.

VOL. III. MARCH, 1860. NO. 3.

### Original Communications.

### ARTICLE 1.

### CASE OF EXTRA UTERINE PREGNANCY, CONTINUING THREE YEARS AND SIX MONTHS—FŒTUS REMOVED BY GASTROTOMY.

REPORTED BY C. GOODBRAKE, M. D., OF CLINTON, ILL.

[Read before the DeWitt County Medical Society, Jan. 8, 1860.]

Mrs. Freize, the subject of the following report, a lady of medium hight, very lean, and about 43 years of age; was brought to my office by her husband, from Piatt county, on the 17th day of last October. The lady informed me that she wished to get my opinion of her case, of which she gave me the following history:

She has had nine children, the youngest about six years old; has been a very stout robust woman, able to do a great deal of hard labor. About April, 1856, she supposed herself pregnant, and in the fifth month she felt the fœtal movements distinctly. She discovered no difference between her then condition and her previous pregnancies; only, that she had more trouble in voiding urine—micturition being very frequent and painful. Some time in the following December, strong bearing down pains came on, and supposing herself in labor, she sent for her family physician, who, judging from her pains, encouraged her with the assurance that her labor would soon be over. In this, however, both the physician and herself were disappointed, for in a few hours her pains gradually subsided, and the doctor after an examination, informed her that her full time had not yet arrived, and left her. She continued to have more or less pains of a bearing down character for two or three weeks, when she again thought herself in labor, and the doctor was again summoned, and after remaining with her a good many hours, he left her as before. From this time on, she continued to have pains of more or less severity; became very anxious about her condition; consulted a great number of physicians, who all differed to some extent in their diagnosis, some of them supposing it to be an ovarian tumor, while others fell in with her own idea of the case, namely, that it was a fœtus. One physician whom she consulted about it three months after the first time she thought herself in labor, prescribed, as she said, some medicine to produce contractions of the uterus, which brought on her menstrual discharge—since which time she has continued to menstruate regularly, up to the time of my seeing her. Her almost constant pains, and her great anxiety about her condition, produced a gradual wasting of the flesh, with diminution of physical strength.—Upon close questioning, she says positively, that there never was any sudden sensation of tearing or giving way in her abdomen, neither had she ever any sudden feeling of faintness or any other symptom which would indicate a rupture of the uterus or Fallopian tube. She also states, that up to the time when she considered herself at full period, and for some time after, her abdomen was elastic and of uniform size; but after that time it became gradually harder and of irregular shape—the bulk of the tumor occupying the right side. She also states, that for some time after her expected confinement, her breasts secreted milk. She informed me that all the physicians she had consulted agreed that no medicine could do her any good, and that if she did not wish to trust to nature for a remedy, she would be compelled to have recourse to surgical aid. And it was very evident that she had fully made up her mind to submit to an operation.

Upon examining the abdomen, I found a large tumor occupying the right side, extending from the illiac fassa, to above the umbilicus, and a little to the left of the linea alba. The tumor felt hard and somewhat irregular, and *appeared* to be movable to some extent.

On examination per vaginum, I ascertained that a round tumor, presenting a round smooth surface, occupied the pelvic cavity. It impinged firmly on the right side, but the finger could be made to pass between it and the wall of the pelvis on the left. The uterus occupied a position behind and to the left of the tumor. I was unable at this examination, although I used my best endeavors, to find the os tincæ. A catheter introduced into the uretha, passed behind the tumor—indicating that both the uterus and bladder were crowded from their true position.

I was not satisfied as to the character of the tumor. At first I was inclined to favor the lady's notions of the case, and believed it to be a case of extra uterine pregnancy, but upon consulting my books, and revolving the case in my own mind, I reasoned myself completely out of that belief, and came to the conclusion that it was most probably a fibro cartilaginous tumor. The woman wishing to know whether I would undertake its removal, I informed her of the *uncertainty* of the nature of her case, of the danger of an operation, and endeavored to prevail upon her to go to Chicago and take the advice of Professors Brainard, Byford and Miller; but she and her husband both answered, that their pecuniary means were not such as to justify them in going to Chicago or Cincinnati; that they had consulted a great number of physicians, that they had been recommended to me by their friends, and they wished me to operate.

I finally agreed to visit her at her residence, on the next Monday; ordered her to take a dose of oil on the Sunday previous, and not to take any breakfast, except a little tea or coffee, on Monday morning. I promised to open the abdomen, and if it was found that the tumor could be removed without endangering her life too much, I would extirpate it; but, if on the other hand, it should prove to be too strongly adherent, or of such a character as that its removal could result in no good to the patient, I would close the wound, leaving the tumor *in situ*. I told the woman to take her case into serious consideration, and if she came to the conclusion not to have the operation performed, to let me know in the mean time, by letter or otherwise.

Accordingly, on Monday, the 24th day of last October, I visited Mrs. F. at her house, accompanied by Drs. Lewis and Tyler of Marion, and Drs. Richards and McHugh of Mount Pleasant; also, Messrs. B. K. Shurtleff and Rolla Richards, medical students. Several of the gentleman present had previously examined the case, but it was deemed advisable to make another thorough examination. At this examination, the mouth of the uterus was found, and we endeavored to introduce the uterine sound, but this was found impracticable on account of the obliquity of the uterus, and the encroachment of the tumor.

The lady, as well as her husband, were again advised of the severity, immediate danger, and ultimate uncertainty of the operation. However, with all these facts before them, they still urged that an operation should be undertaken.

In view of this determination on their part, the preliminary arrangements were made. The atmosphere of the room was kept moist by the evaporation of water from kettles on the stove. An artificial serum was prepared according to Doctor Peaslee's formula;* and all preparations made that were deemed necessary. The woman was placed on a table, the head and shoulders raised, a sheet applied as a diaper, and the operation performed in presence of the gentlemen already named, who kindly assisted by their council.

The patient being under the influence of a mixture, of one part chloroform to four of sulphuric ether, I made an incision in the linea alba, about four inches in length, down to the peritoneum, no hæmorrhage occurring, I cut down through it also. I now introduced my hand, after immersing it in the artificial serum, and soon satisfied myself that it was actually a fœtus enveloped in a sack of its own. The sack was found firmly adherent in the right illiac fassa, and to a considerable

* Amer. Jour. Med. Sciences. Vol. XXXVI, Page 895.

extent, to the prietal peritoneum on the right side. There were no adhesions anteriorly, nor to the intestines, which were all crowded to the left side. This diagnosis was confirmed by Dr. McHugh, who also made a thorough examination. In order to ascertain the condition of the fœtus, a small incision was made in the sack, when it was found in a pretty good state of preservation; and upon a hurried consultation, it was deemed advisable to remove it, and as much of its sack as practicable.

The incision was now extended upwards as far as the umbilicus, and down to within an inch of the pubes. The incision through the sack was also enlarged, when the fœtus was removed with great difficulty, owing to the strong adhesions between it and the sack. When the fœtus was lifted out, the cord was found to be yet entire and attached to a very small placenta of a cartilaginous character, low down in the pelvis. The placenta was located immediately over the space where the sack also adhered to the broad ligament. The uterus was a little enlarged, but otherwise it seemed in a normal condition. The cord, as much of the placenta and sack as could be got away without lacerating the peretoneum, was now removed, the parts carefully sponged, and the incision brought neatly together by the interrupted suture, supported by adhesive strips; and the dressing finished by the compress and bandage. The patient rallied from the effects of the anesthetic about the time the dressing was completed, and was placed snugly in bed, a dose of laudanum was administered, and she expressed herself as quite comfortable. Her pulse was good.

The time occupied in bringing her under the influence of the anesthetic, in the operation, and until she was placed in bed, was 45 minutes, as observed by Mr. Shurtleff. It was estimated that there was not over an ounce of blood lost during the operation.

The patient was left, according to previous arrangement, in the care of Drs. Richards and McHugh; one of them remaining the first night at her house. They visited her regularly afterwards, twice a day. The bladder was evacuated morning and evening, and opium and brandy administered according to indications.

The physicians reported that the patient did quite well for the first 48 hours; after which she became restless, her pulse grew gradually weaker and more frequent, until it became imperceptible at the wrist.

Dr. McHugh informed me that the wound looked well, and that there was no swelling of the abdomen up to the time of her death, which occurred on the fifth day after the operation..

A post-mortem examination was solicited, but was refused by the friends.

The fœtus, which I presented to the Obstetrical Museum of the Rush Medical College, is of female sex, of medium size as at full period; well developed; nails on fingers and toes well grown; weight not ascertained. The position it occupied in the abdomen was as follows:—The thighs were flexed on its abdomen, and the legs flexed on the thighs, with the face doubled low down between the knees, and it was kept in this position by the strong adhesions between these several parts. The head rested in the right illiac fassa, and the breech, being to the left of the head, passed down, to some extent, behind the pubic bone, displacing the uterus and bladder as before described. The only mark of decomposition observable on the fœtus, was on the side, where a spot about twice the size of a dollar had sloughed out, exposing the ribs and some of the internal organs.

Remarks.—The question may with great propriety be asked—was it right and proper to operate, in the case of Mrs. F.? I would answer, that it would certainly have been better for the woman if she had not submitted to the operation; as she might, probably, have lived several years. Though this is only a probability; for where the sloughing had commenced on the fœtus, there was no adhesion, neither between it and the sack, nor between the sack and the walls of the abdomen. So that if decomposition of the fœtus had advanced, the woman must have died. And even in a large majority of the cases on record, where adhesions have taken place, and where the sloughs have found their way through the walls of the abdomen, the patients died nevertheless.

If, when I opened the abdomen, I had found a tumor as strongly adherent as was the fœtal sack, I would most certainly have desisted, and closed the wound, according to my promise to the patient. But finding a fœtus, and the edges of the wound retracting strongly, it was deemed impossible for the wound to heal before fœtal decomposition would have set in, which would have made a very bad case of it indeed.

